# Involvement of the flagellar assembly pathway in *Vibrio alginolyticus* adhesion under environmental stresses

**DOI:** 10.3389/fcimb.2015.00059

**Published:** 2015-08-12

**Authors:** Lu Wang, Lixing Huang, Yongquan Su, Yingxue Qin, Wendi Kong, Ying Ma, Xiaojin Xu, Mao Lin, Jiang Zheng, Qingpi Yan

**Affiliations:** ^1^Key Laboratory of Healthy Mariculture for the East China Sea, Ministry of Agriculture, Fisheries College, Jimei UniversityXiamen, China; ^2^College of Ocean and Earth Sciences, Xiamen UniversityXiamen, China

**Keywords:** *Vibrio alginolyticus*, adhesion, flagellar assembly pathway, environmental stresses

## Abstract

Adhesion is an important virulence factor of *Vibrio alginolyticus*. This factor may be affected by environmental conditions; however, its molecular mechanism remains unclear. In our previous research, adhesion deficient strains were obtained by culturing *V. alginolyticus* under stresses including Cu, Pb, Hg, and low pH. With RNA-seq and bioinformatics analysis, we found that all of these stress treatments significantly affected the flagellar assembly pathway, which may play an important role in *V. alginolyticus* adhesion. Therefore, we hypothesized that the environmental stresses of the flagellar assembly pathway may be one way in which environmental conditions affect adhesion. To verify our hypothesis, a bioinformatics analysis, QPCR, RNAi, *in vitro* adhesion assay and motility assay were performed. Our results indicated that (1) the flagellar assembly pathway was sensitive to environmental stresses, (2) the flagellar assembly pathway played an important role in *V. alginolyticus* adhesion, and (3) motility is not the only way in which the flagellar assembly pathway affects adhesion.

## Introduction

*Vibrio alginolyticus* is one of the most important opportunistic pathogens and has been associated with several diseases of cultivated marine animals, including those in fish and shellfish (Heo et al., 2012), shrimp (Ahmed et al., 2015) and those associated with coral reefs (Xie et al., 2013). *V. alginolyticus* has caused mass mortality of cultured large yellow croakers (*Pseudosciaena crocea*) and has led to considerable economic losses.

Adhesion to fish tissue surfaces is one of the most important steps in the initial stage of *V. alginolyticus* infection (Snoussi et al., 2009). Adhesion to the mucus covering the intestine and skin is a critical step for host colonization and the infection of pathogenic bacteria. Therefore, adhesion is a crucial bacterial virulence factor and attracts widespread attention. Several genes have been shown to be associated with bacterial adhesion (Vayssier-Taussat et al., 2010; Paing and Tolia, 2014; Yang et al., 2014); however, the molecular mechanism of *V. alginolyticus* adhesion remains unclear.

The capability of bacterial adhesion could be substantially affected by environmental factors (Yan et al., 2007). pH, an important environmental factor, has a substantial influence on bacterial attachment. Balebona et al. (1995) found optimum adhesion of *Vibrio* strains to the skin mucus of *Sparus aurata* at pH 8.1. Metals are introduced into the environment from various sources, such as from industrial processes and during the mining and refining of metal ores. The rapid expansion of industry and increases in domestic activities in the last century has induced a concomitant increase in the levels of metals released into the environment (Xiao et al., 2015). The biochemical and physiological mechanisms whereby metals exert their effects on microorganisms have been reviewed (Haferburg and Kothe, 2007). According to our previous results, we found that Cu, Pb, Hg, and low pH could reduce the adhesion ability of *V. alginolyticus*, whereas high pH, low salinity, high salinity, low temperature, and high temperature did not significantly affect adhesion. The way in which the adhesion process was influenced by environmental factors was also unclear.

RNA-seq has been widely used in bacterial transcriptome profiling and could be used to determine the expression levels of large numbers of genes in a single experiment in an attempt to obtain insight into the molecular biological mechanisms in bacteria (Mandlik et al., 2011). In our previous research, we presented the RNA-seq in *V. alginolyticus* cultured under stress conditions (including Cu, Pb, Hg, and low pH) and normal conditions to obtain new clues for the mechanism(s) underlying adhesion regulation in *V. alginolyticus*. Using RNA-seq and bioinformatics analysis, we found that all of these stress treatments could significantly affect the flagellar assembly pathway (Kong et al., 2015).

The bacterial flagellum is an organelle for cell propulsion, which is composed of three parts: basal-body, hook, and filament. Flagellar assembly is a logistical process which needs thousands of proteins subunits export for long distances, except for the MS ring, part of the basal body, which is located in the membrane. Other subunits have to be exported through the type III flagellar export system and accomplish assembly by order without the help of anything but themselves.

Flagellar assembly begins with the MS ring encoded by *FliF*, followed by the assembly of C-ring and type III export apparatus. C-ring is comprised of three components encoded by *FliG, FliM, FliN*, which acts as a motor/switch of motor force generators. Plus, motor force generators include the products of *MotA* and *MotB*, which act as stator. Type III export apparatus are encoded by 9 genes, *FlhA, FlhB, FliO, FliP, FliQ* and *FliR, FliH, FliI*, and *FliJ*, respectively, through which protein subunits are exported to the assembly place (Minamino et al., 2000). Next step is the assembly of rod, which includes proximal rod and distal rod, encoded by *FlgF* and *FlgG*. After the rod assembly is the elongation of hook, which is controlled by *FlgE, FliK*, and *FlgD*. Of the above three genes, *FliK* is responsible for the flagellar length. Once the hook elongation completes, late gene products will be secreted through type III export apparatus. The last step is the assembly of filament encoded by *FliC* (Kubori et al., 1992).

The flagellar assembly pathway may play an important role in the adhesion process of *V. alginolyticus.* Therefore, we hypothesized that the sensitivity to environmental stresses of the flagellar assembly pathway may be a way in which environmental conditions affect adhesion.

The aim of this study was to investigate (1) the relationship between the flagellar assembly pathway and *V. alginolyticus* adhesion, and (2) the sensitivity of the flagellar assembly pathway to environmental stresses.

## Materials and methods

### Functional classification and enrichment analysis for differential expression genes (DEGS)

For DEGs annotation, we used the Blast2GO program to obtain Gene Ontology (GO) annotation of the unigenes. After acquiring the GO annotation for every gene, we used WEGO software to carry out GO functional classification for all genes and understand the distribution of gene functions of the species at the macro level. The calculated *P*-value went through Bonferroni Correction, taking corrected *P* ≤ 0.05 as a threshold. GO terms fulfilling this condition were defined as significantly enriched GO terms in DEGs.

The KEGG pathways annotation was carried out using Blastall software against KEGG (http://www.genome.jp/kegg/) database. *Q*-value was defined to be the FDR analog of the *P*-value. Pathways with *Q*≤0.05 were regarded as significantly enriched in DEGs.

### Bacterial strain and culture conditions

Pathogenic *V. alginolyticus* (ND-01) was isolated from naturally infected large yellow croakers and previously confirmed as a pathogen by our lab (Kong et al., 2015). Physiological saline with 10% glycerol was used to store the sample at −80°C. Tryptic soy agar (TSA) supplemented with 2% NaCl was used to maintain the *V. alginolyticus* at 28°C, while Luria–Bertani (LB) broth supplemented with 2% NaCl was used to grow the *V. alginolyticus* with shaking (220 r.p.m.). To validate the results of the RNA-seq, the *V. alginolyticus* were stressed by exposure to Cu (50 mg/L), Pb (100 mg/L), Hg (50 mg/L), and low pH (HCl was used to lower the pH to 5), which reduced the adhesion ability of *V. alginolyticus*. LB broth (supplemented with 2% NaCl, pH = 7) was used to culture the control group. There were six replicates for each treatment.

*Escherichia coli* SM10 was bought from TransGen Biotech (China). Growth in LB broth (220 r.p.m., 37°C) or on LB plates at 37°C was used.

### Transient gene silencing

Short interfering RNA (siRNA) (21–23 nt) with a characteristic and highly specific 2-3-nucleotide 3′ overhang was synthesized according to the gene sequence by GenePharma Co. Ltd. (Shanghai, China). Negative control siRNA and treatment siRNA sequences are listed in Table S1.

The electro transformation of *V. alginolyticus* strains was carried out using a modified method by Lancashire et al. (2005). For viable cell preparation, *V. alginolyticus* in a stationary phase was 1% (v/v) inoculated into 5 ml fresh LB medium until an OD_600_ of 0.3–0.5 was reached. The bacteria were collected via centrifugation (4000 g, 4°C, 10 min), and the pellet was washed twice in ice-cold sterile water and a third time in ice-cold 10% glycerol in water (v/v). Then, the cells were resuspended in 1 ml 10% glycerol.

Electroporation was carried out using a Bio-Rad MicroPulser (Bio-Rad Laboratories, Inc.) according to our previous study (Huang et al., 2015). A total of 100 μl of competent cells was mixed with 2 μl of siRNA (20 μM). The cells and siRNA were transferred to the cuvette after being stored on ice for 30 min. Following electroporation (1.8 KV, 6 ms), 900 μl of LB medium was added immediately and then incubated at 28°C for 1, 6, 12, and 24 h prior to RNA extraction and RT-PCR.

*V. alginolyticus* with RNAi treatments displayed significant silencing at 1–6 h (mentioned below). Thus, the *V. alginolyticus* cells were recovered at 28°C and 50 r.p.m. for 2 h after electroporation to conduct the *in vitro* adhesion assay as described below.

### Stable gene silencing

The stable gene silencing was performed according to other research (Darsigny et al., 2010). Competent bacteria cells were collected as mentioned above. Four short hairpin RNA sequences targeting the coding region of *FliC, FliD, FliS*, and *FlgH* mRNA were designed by Shanghai Generay Biotech Co., Ltd (Shanghai, China) (Table S2). The annealed oligo nucleotides were ligated with pACYC184 vectors, which were harvested from *Bam*HI and *Sph*I double digestion via T4 DNA ligase (TaKaRa, Japan) according to the manufacturer's recommendations. The recombinant pACYC184 vectors were transformed into SM10 via heat shock and then transferred via conjugation from SM10 to *V. alginolyticus*. Empty pACYC184 vector was used as a control. Chloramphenicol (34 μg/ml) was used to screen the stable silenced clones, which were used for RNA extracts and *in vitro* adhesion and motility assays. The bacteria were inoculated into test tubes filled with semi-soft medium and incubated at 28°C for 6 h to observe motility.

### Transmission electron microscope observation

Formvar-coated grids were floated on 20 μl drops of bacterial cell suspensions. Excess sample was withdrawn by touching the edge of the grid to a cut edge of Whatman filter paper. The grids were negatively stained with a 1% solution of Phosphotungstic acid and observed with a Tecnai F20 (Philips) transmission electron microscope. More than 20 fields of view were observed for each group. The diameter of the flagella were measured using Osiris 4.0 software from images (*n* = 6 cells per condition).

### RNA extraction and reverse transcription

Total RNA was extracted from the bacteria using TRIzol (Invitrogen, Carlsbad, CA) according to the manufacturer's protocol. First-strand cDNA was synthesized from 2 mg of total RNA using a Revert Aid Mu-MLV cDNA synthesis kit according to the manufacturer's protocol.

### Quantitative RT-PCR assay

The expression levels of DEGs of the flagellar assembly pathway identified via RNA-seq were verified using quantitative real-time PCR (QPCR) and the Power SYBR Green PCR Master Mix (AppliedBiosystems) in accordance with the manufacturer's instructions. The expression levels were normalized with 16S RNA (which showed an invariant expression under the experimental conditions in Figure S1), which was calculated using the means of the 2^−ΔΔCt^ method (*n* = 6). The primers are listed in Table S3.

### Mucus preparation

This study was carried out in strict accordance with the recommendations in the Guide for the Care and Use of Laboratory Animals of the National Institutes of Health. The protocol was approved by the Committee on the Ethics of Animal Experiments of the Animal Ethics Committee of Xiamen University (Acceptance No.: XMULAC20120030).

Healthy large yellow croakers caught by commercial fishermen from marine cultured cages in the city of Ningde in the Fujian province of China were used for mucus preparation via our previous method (Kong et al., 2015). The fishes were killed by concussion of the brain by striking the cranium. After washing with sterile PBS (0.01 mol/L pH 7.2), the skin mucus was harvested by scraping off the surface of the skin with a plastic spatula to remove the mucus gel layer; this was homogenized in PBS. The mucus preparations were centrifuged twice (20,000 g, 4°C, 30 min) to remove particulate materials and then filtered through 0.45 and 0.22 μm pore size filters. The mucus samples were adjusted to 1 mg protein/mL PBS using the method of Bradford (Bradford, 1976).

### *In vitro* adhesion assay

The bacterial adhesion assay was performed using our previously described method (Yan et al., 2006). A total of 50 μL of mucus was evenly spread on a 22 × 22 mm glass slide area and fixed with methanol for 20 min. Then, 1 ml of bacterial suspension (10^8^ CFU/ml) was placed on the mucus-coated glass slides, incubated for 2 h at 25°C in a humidified chamber, and washed with PBS five times. Lastly, the bacteria were fixed using 4% methanol for 30 min, dyed via crystal violet for 3 min, and counted under a microscope (× 1000). Each group was conducted in three trials, and 20 fields of view were selected. Negative control was performed using PBS instead of bacterial suspension.

### Soft agar plate motility assay

For the assay of motility of *V. alginolyticus* strains, the soft agar method was adapted. Overnight cultures were diluted to OD_660_ = 0.03. A 1 μl drop of the cell suspension was spotted on to the center of the LB plates (0.3% agar) at 37°C for 20 h. The diameters of the colonies were measured at 20 h.

### Data processing

The results are reported as the means ± S.E. The data were statistically analyzed with One-Way ANOVA followed by Dunnett's multiple comparison test via SPSS 13.0 software. A value of *P* < 0.05 was used to indicate significant differences.

## Results

### RNA-seq screening for differentially expressed genes

Low pH reduced adhesion to 43.42% of the unstressed control, while Cu, Pb, and Hg reduced adhesion to 62.59, 60.74, and 59.35% of the unstressed control, respectively. Therefore, we presented the first RNA-seq in *V. alginolyticus* cultured under stress conditions, including Cu, Pb, Hg, low pH, as well as normal conditions. The data were deposited in the NCBI Sequence Read Archive (SRA) under accession number SRP049226.

RNA-seq and DEGs analysis finally yielded 1637, 1085, 846, and 1791 DEGs in the Cu-, Pb-, Hg-, and low pH-treated groups compared with the control group, respectively. Analysis of GO categories showed that the functional distribution of the DEGs from each stressed group was similar. In the libraries, most of the corresponding biological process genes were involved in cellular processes, metabolic processes, establishment of localization and localization. Most of the cellular component genes encoded proteins associated with cell, cell part, membrane and membrane part; and most of the molecular function genes were associated with binding and catalytic activity. Using KEGG, DEGs were assigned to 164 KEGG pathways. Those pathways with the greatest representation by DEGs were “ABC transportersystem,” “Two-component system,” “Glyoxylate and dicarboxylate metabolism,” and “Flagellar assembly.” These annotations provide a substantial resource for investigating specific processes, functions and pathways during bacteria adherence. Among the 164 KEGG pathways, some pathways are known to be closely related to adhesion, for example: “Two-component system,” “Bacterial chemotaxis,” and “Flagellar assembly.”

The flagellar assembly pathway was selected for further research because it was closely related to adhesion. A GO functional analysis and KEGG Pathway analysis showed that 36 significantly differentially expressed genes with products involved in structural and regulatory proteins were associated with the flagellar assembly pathway. The type and number of DEGs in the flagellar assembly pathway vary across stressed conditions (Figures S2–S5). There were 4 commonly down-regulated DEGs: *FliD, FliC, FlgH*, and *FliS*. *FlgH* encodes the L ring, whereas *FliC* and *FliD* encode the filament and filament cap with the product of *FliS* working as a chaperone. These genes were significantly differentially expressed in all stress groups; thus, they may be the genes that are most sensitive to environmental stresses. *FliC* displayed the largest declines for all stressed groups (Table 1). Therefore, *FliC* appears to be the most sensitive to all four stress conditions.

**Table 1:** **Fold change of commonly down-regulated DEGs of Flagellar Assembly Pathway identified by RNA-seq**.

	**Cu**	**Pb**	**Hg**	**Low pH**
FliD	−8.34	−6.92	−5.82	−2.14
FliC	−34.78	−15.24	−17.88	−2.87
FlgH	−7.16	−6.02	−4.26	−2.50
FliS	−9.13	−5.86	−4.86	−2.53

### Validation of the results of high through-put sequencing

**Figure 1:**
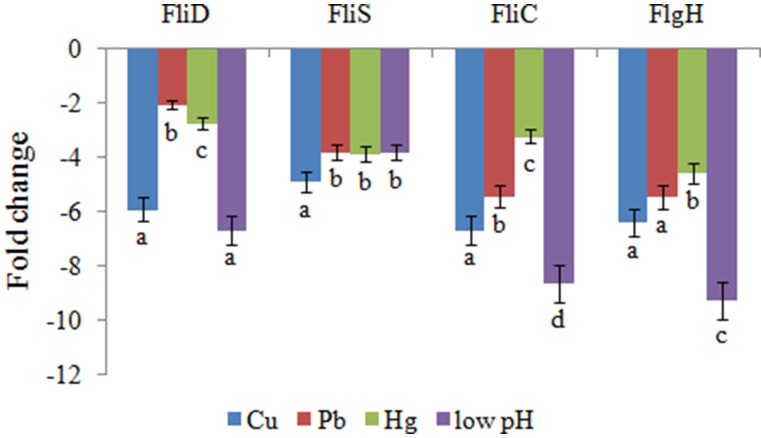
**QPCR analysis of the expression of *FliD, FliC, FlgH*, and *FliS* after stress treatments in comparison to untreated control**. The data are presented as the means ± S.D. (*n* = 6). The means of the treatments not sharing a common letter are significantly different at *P* < 0.05.

QPCR was performed on these four genes to validate the RNA-seq results. The QPCR results matched those of the sequencing: the Cu, Pb, Hg, and low pH treatments significantly down-regulated the expression of *FliD* (by 5.9-, 2.1-, 2.7-, and 6.7-fold, respectively), *FliC* (by 6.7-, 5.4-, 3.2-, and 8.6-fold, respectively), *FlgH* (by 6.4-, 5.5-, 4.6-, and 9.2-fold, respectively), and *FliS* (by 4.9-, 3.8-, 3.9-, and 3.8-fold, respectively) (Figure 1). These reinforced the reliability of the sequencing data.

### Effects of transient gene silencing

*V. alginolyticus* was treated with siRNAs, and the expression levels of the target genes were assessed after 1, 6, 12, and 24 h.

**Figure 2:**
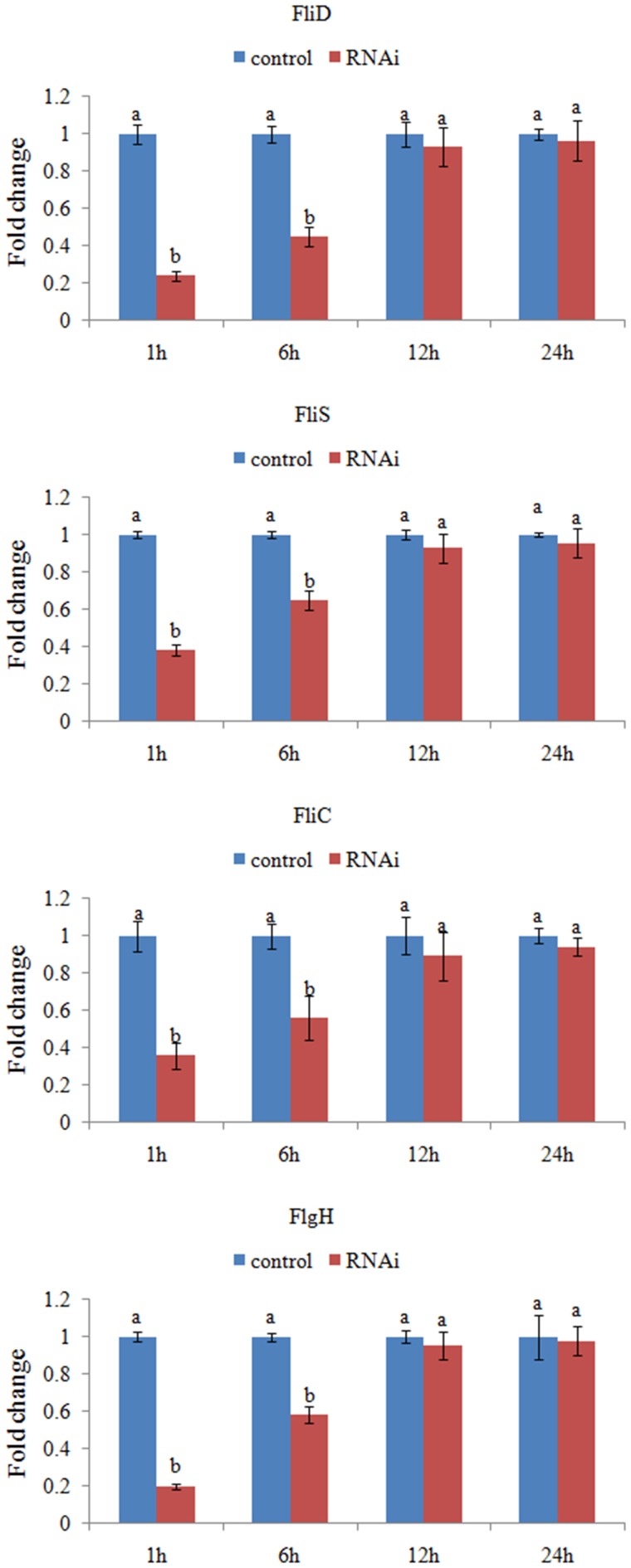
**QPCR analysis of the expression of *FliD, FliC, FlgH*, and *FliS* after transient gene silencing at 1, 6, 12, and 24 h in comparison to the control**. The data are presented as the means ± S.D. (*n* = 6). The means of the treatments not sharing a common letter are significantly different at *P* < 0.05.

The expression levels of these target genes with siRNA treatments were normalized against the corresponding control (scrambled) siRNA treatments. Based on the percentage of expression decrease (Figure 2), *V. alginolyticus* with siRNA treatments displayed significant (*p* < 0.05) gene silencing at 1–6 h, whereas siRNAs were not effective in *V. alginolyticus* at any of the time points after 6 h. The reduction in the target genes indicated that the siRNAs worked.

**Figure 3:**
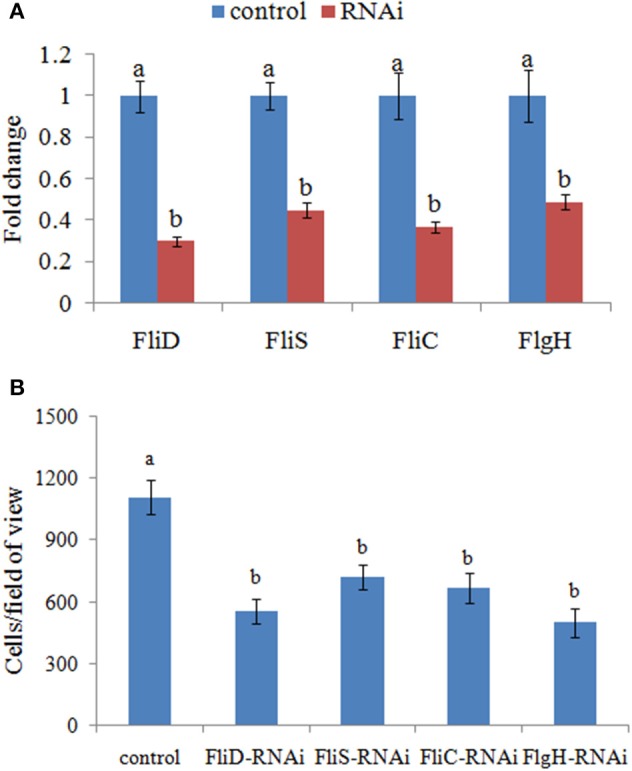
**Transient RNAi reduced the adhesion of *V. alginolyticus*. (A)** QPCR analysis of the expression of *FliD, FliC, FlgH*, and *FliS* after transient gene silencing at 2 h in comparison to the control. The data are presented as the means ± S.D. (*n* = 6). The means of the treatments not sharing a common letter are significantly different at *P* < 0.05. **(B)** The adhesion capacity to mucus of transient silenced *V. alginolyticus* at 2 h. The data are presented as the means ± S.D. (*n* = 3). The means of the treatments not sharing a common letter are significantly different at *P* < 0.05, as assessed using One-Way ANOVA followed by Dunnett's test.

Therefore, the adhesion ability of *V. alginolyticus* under normal conditions and RNAi conditions was compared at 2 h. QPCR analysis of the expression of *FliD, FliC, FlgH*, and *FliS* after transient gene silencing was performed at 2 h, in order to further ensure that they were significantly repressed at 2 h (Figure 3A). *In vitro* adhesion assay showed that approximately 1108 cells/view of the control *V. alginolyticus* adhered to the slides, whereas the numbers of adherent bacterial cells of *FliD, FliC, FlgH*, and *FliS*-RNAi *V. alginolyticus* were 554, 665, 499, and 721 cells/view, respectively (Figure 3B), which demonstrated that the adhesion ability of *V. alginolyticus* under RNAi conditions was significantly impaired.

### Effects of stable gene silencing

**Figure 4:**
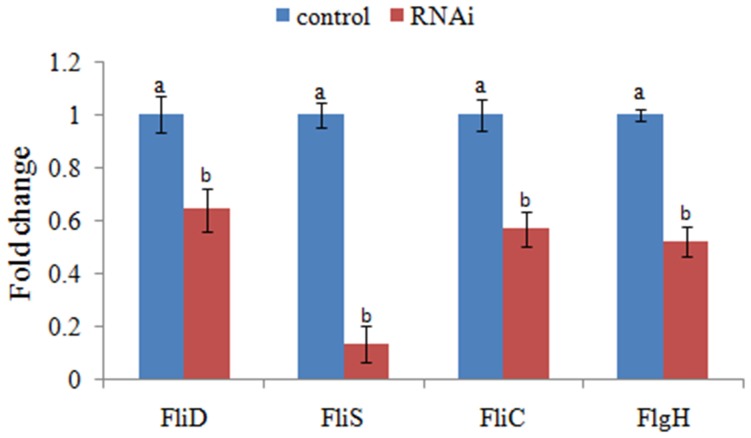
**QPCR analysis of the expression of *FliD, FliC, FlgH*, and *FliS* after stable gene silencing in comparison to the control**. The data are presented as the means ± S.D. (*n* = 6). The means of the treatments not sharing a common letter are significantly different at *P* < 0.05.

The expression levels of *FliD, FliC, FlgH*, and *FliS* were significantly reduced in stable silenced clones by 1.6-, 1.8-, 1.9-, and 7.5-fold, respectively (Figure 4). These results reinforced the reliability of stable gene silencing.

**Figure 5:**
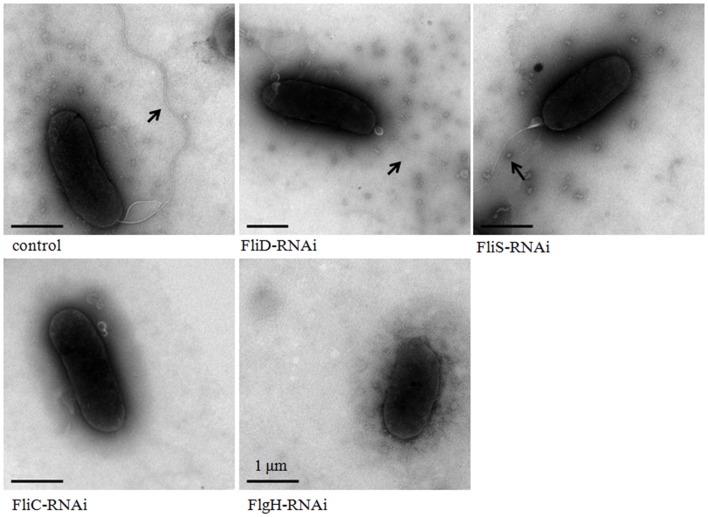
**Transmission electron micrographs of stable silenced *V. alginolyticus* strains**. The arrow indicates the flagellum.

The flagella of stable silenced *V. alginolyticus* strains were examined by transmission electron microscope (Figure 5). These micrographs showed that flagella were presented on the cells surface of control, FliD-RNAi and FliS-RNAi strain, while the flagella of FliD-RNAi and FliS-RNAi strain were significantly thinner than the control (*p* < 0.05). The diameters of the flagella of control, FliD-RNAi and FliS-RNAi strain were 0.018 ± 0.0032, 0.010 ± 0.0017, and 0.011 ± 0.0013 μm, respectively. Meanwhile, no flagella were observed on the cell surface of the FliC-RNAi and FlgH-RNAi strain.

**Figure 6:**
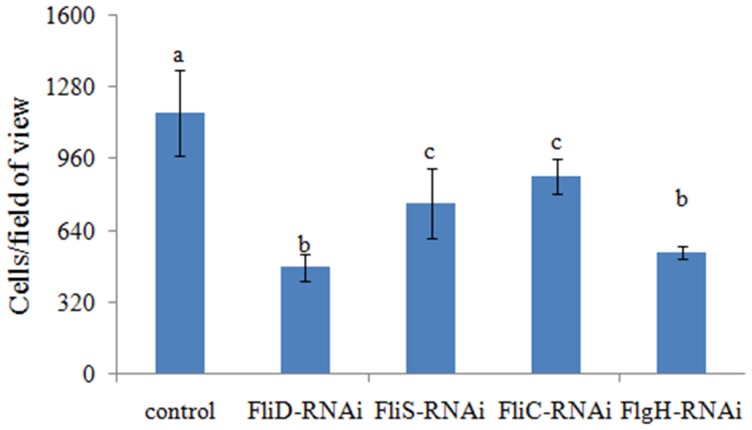
**The adhesion capacity of stable silenced *V. alginolyticus* to mucus**. The data are presented as the means ± S.D. (*n* = 3). The means of the treatments not sharing a common letter are significantly different at *P* < 0.05, as assessed using One-Way ANOVA followed by Dunnett's test.

The adhesion ability of the stable silenced clones was also detected. Our results showed that approximately 1165 cells/view of the control *V. alginolyticus* adhered to the slides, whereas the numbers of adherent bacteria of *FliD, FliC, FlgH*, and *FliS*-RNAi *V. alginolyticus* were 476, 881, 541, and 761 cells/view, respectively (Figure 6), which demonstrated that stable silenced clones were significantly impaired in their adhesion ability.

**Figure 7:**
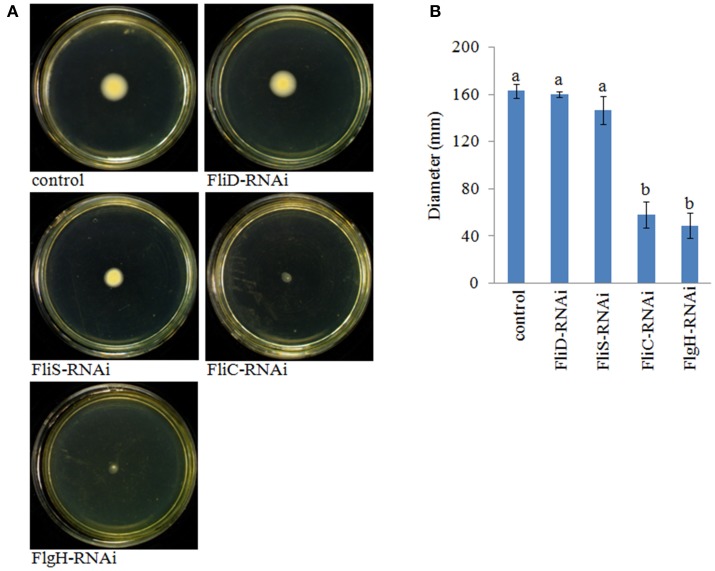
**The motility behavior on soft agar plates of stable silenced *V. alginolyticus* strains**. **(A)** Typical images of spreading of stable silenced *V. alginolyticus* strains and the control. **(B)** Diameter of the colony of each strain. The data are presented as the means ± S.D. (*n* = 3). The means of the treatments not sharing a common letter are significantly different at *P* < 0.05, as assessed using One-Way ANOVA followed by Dunnett's test.

The motility of the stable silenced clones was assessed. Our results showed that *FliD*-RNAi and *FliS*-RNAi *V. alginolyticus* exhibited no change in motility, whereas *FliC*-RNAi and *FlgH*-RNAi *V. alginolyticus* displayed decreased motility compared with the control group (Figure 7).

## Discussion

Adhesion to mucus is essential for infection by pathogenic bacteria (Snoussi et al., 2009). Therefore, the inhibition of bacterial adhesion to the host may have therapeutic value (Acord et al., 2005). Bacterial adhesion could be substantially influenced by environmental factors (Yan et al., 2007); however, little is known about how environmental factors affect the adhesion process. Thus, it is important to understand the mechanisms underlying the adhesion of bacteria and the effects of environmental factors, which may assist in developing preventive measures for reducing infection.

The adhesion of *V. alginolyticus* to glass surfaces is dependent on swimming speed, and a higher speed results in higher adhesion (Kogure et al., 1998); however, bacteria motility depends on flagellum biogenesis and assembly. In our previous study, we analyzed the effects of environmental factors (such as Cu, Pb, Hg, high pH, low pH, high salinity, low salinity, high temperature, and low temperature) (Table S4) on *V. alginolyticus* adhesion. We found that Cu, Pb, Hg, and low pH could significantly reduce the adhesion ability of *V. alginolyticus.* The present study showed that Cu, Pb, Hg, and low pH could affect the flagellar assembly pathway. A GO functional analysis and KEGG Pathway analysis yielded 36 significantly differentially expressed genes with products that involved structural and regulatory proteins to be associated with the flagellar assembly pathway. These results demonstrated the relationship between the flagellar assembly pathway and the adhesion process of *V. alginolyticus.* Furthermore, we hypothesized that the sensitivity to environmental stresses of the flagellar assembly pathway may be one way in which environmental conditions affect adhesion.

The type and number of DEGs in the flagellar assembly pathway vary across stressed conditions. There were 4 commonly down-regulated DEGs: *FliD, FliC, FlgH*, and *FliS*. These genes significantly differentially expressed in all stress groups and thus may be those that are the most sensitive to environmental stresses.

*FlgH* encodes the L-ringin the lipid outer cell membrane through which the axial filament (rod, hook, and flagellum) passes. The last step of the flagellar assembly is the assembly of the filament encoded by *FliC* (Kubori et al., 1992). During flagellar assembly, both the filament and hook have cap proteins. The filament cap, which is encoded by *FliD*, promotes assembly in a similar manner to the chaperone (Yonekura et al., 2000). The hook cap protein encoded by *FlgD* functions in the same manner. The flagellar assembly proteins are divided into early and late gene products according to their orders in the process. *FlhC* and *FlhD* are required for the early gene products, whereas *FlgM* inhibits late gene products prior to their need. Many late gene products have chaperones because they spend more time in the cytoplasm. Hook-filament junction proteins, flagellin, filament capping protein and P-ring are associated with *FlgN, FliS, FliT* (Bennett and Hughes, 2000; Auvray et al., 2001; Bennett et al., 2001), and *FlgA*, respectively (Nambu and Kutsukake, 2000). Moreover, *FliJ* interacts with *FliH* and *FlhA* (Fraser et al., 2003). Thus, these chaperones prevent the degradation and aggregation of substrates presumably by interacting with them to prevent mis-folding (Minamino et al., 2000). The filament and filament cap were the final performers in motility. The perturbation of the assembly of the L-ring, the filament and its cap decrease the rotation of the motor, thereby reducing the ability to move in an attractant and repellent environment. Therefore, *FliD, FliC, FlgH*, and *FliS* are very important and may affect bacterial adhesion via motility.

To verify our hypothesis that the sensitivity to environmental stresses of flagellar assembly pathway may be a way in which environmental conditions affect adhesion, we examined the relationship between *FliD, FliC, FlgH*, and *FliS* and adhesion via QPCR, RNAi, and adhesion and motility assays. Our results indicated that *FliD, FliC, FlgH*, and *FliS* are closely related to adhesion, which reinforced the bioinformatics results and supported our hypothesis. However, the results of the motility assay showed that not all of the four *V. alginolyticus* strains after stable gene silencing were deficient in motility. *FliD*-RNAi and *FliS*-RNAi *V. alginolyticus* exhibited no change in motility, whereas *FliC*-RNAi and *FlgH*-RNAi *V. alginolyticus* displayed decreased motility. Therefore, adhesion is closely related with motility, but motility is not the only avenue through which the flagellar assembly pathway affects adhesion. Further research is necessary.

In traditional functional gene research, non-directional knockout is conducted via transposon mutagenesis, which features non-direction and a bias in the screening procedure (Autret and Charbit, 2005). Therefore, transposon mutagenesis is demanding and the aim is unclear. In this study, we adopted a comparative transcriptome analysis for the high-throughput screening of DEGs related with adhesion, along with RNAi and an *in vitro* adhesion assay and motility assay for validation. Due to its high accuracy and efficiency, this method can provide a reference for mining and researching other functional genes.

In conclusion, our results indicated that the flagellar assembly pathway was sensitive to environmental stresses and played a key role in the adhesion process of *V. alginolyticus* and that motility is not the only avenue through which the flagellar assembly pathway affected adhesion. Furthermore, because the flagellar assembly pathway was sensitive to environmental stresses, it could be considered to be the basis of a strategy for therapeutic interventions in bacterial pathogenicity.

## Conflict of interest statement

The authors declare that the research was conducted in the absence of any commercial or financial relationships that could be construed as a potential conflict of interest.

## Supplementary material

Figure S1 **Expression stability assay of the references genes**. GeNorm analysis uses the delta Cq method, with the PCR efficiencies based on a dilution series of pooled cDNA samples, and the internal control gene-stability measure M is defined as the average pairwise variation of a special gene with all other control genes. Genes have the most stable expression are marked with the lowest *M*-values, and then exclude the gene with the highest *M*-value step by step to gain a combination of two constitutively expressed housekeeping genes that have the most stable expression in the tested samples.

Figure S2 **The overall scheme for genes involved in the flagellar assembly pathway that were significantly affected by Cu stress**. Green and red indicate decreased and increased expression, respectively.

Figure S3 **The overall scheme for genes involved in the flagellar assembly pathway that were significantly affected by Pb stress**. Green and red indicate decreased and increased expression, respectively.

Figure S4 **The overall scheme for genes involved in the flagellar assembly pathway that were significantly affected by Hg stress**. Green and red indicate decreased and increased expression, respectively.

Figure S5 **The overall scheme for genes involved in the flagellar assembly pathway that were significantly affected by low pH stress**. Green and red indicate decreased and increased expression, respectively.
