# Supplementary figures and images for: Involvement of the flagellar assembly pathway in Vibrio alginolyticus adhesion under environmental stresses
Source: Front Cell Infect Microbiol. 2015 Aug 12;5:59. doi: 10.3389/fcimb.2015.00059 (PMC4533019; doi:10.3389/fcimb.2015.00059)

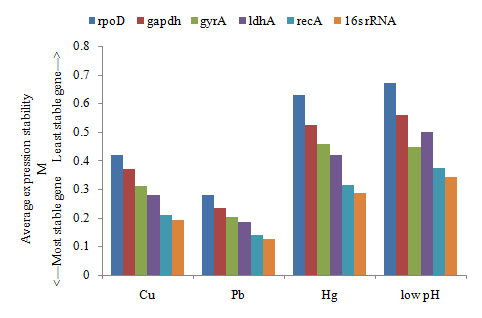

Supplement: Figure S1 — Expression stability assay of the references genes. GeNorm analysis uses the delta Cq method, with the PCR efficiencies based on a dilution series of pooled cDNA samples, and the internal control gene-stability measure M is defined as the average pairwise variation of a special gene with all other control genes. Genes have the most stable expression are marked with the lowest M-values, and then exclude the gene with the highest M-value step by step to gain a combination of two constitutively expressed housekeeping genes that have the most stable expression in the tested samples. [file Image1.TIF]

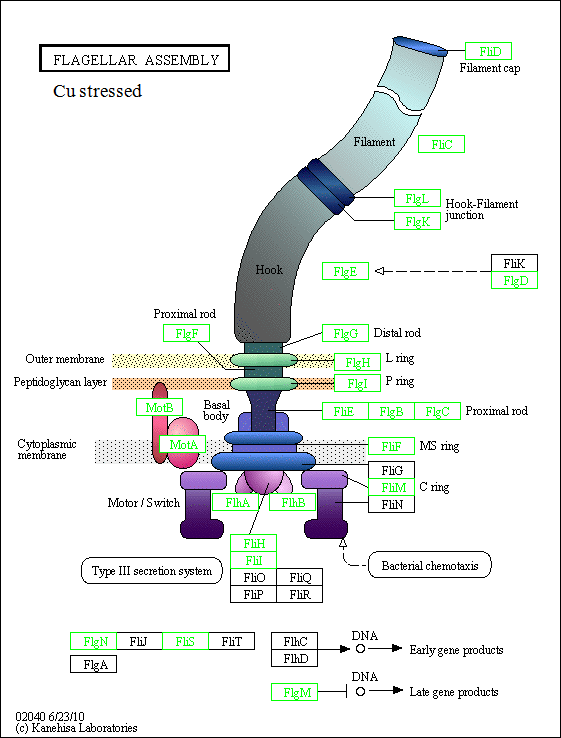

Supplement: Figure S2 — The overall scheme for genes involved in the flagellar assembly pathway that were significantly affected by Cu stress. Green and red indicate decreased and increased expression, respectively. [file Image2.TIF]

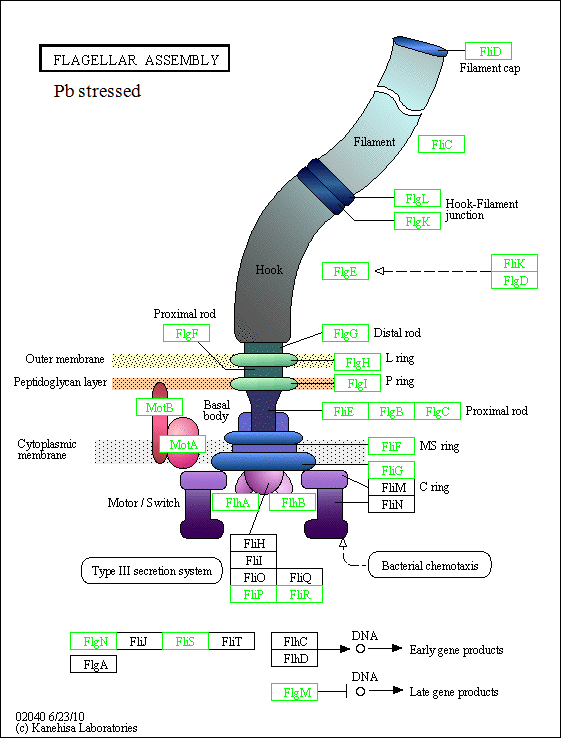

Supplement: Figure S3 — The overall scheme for genes involved in the flagellar assembly pathway that were significantly affected by Pb stress. Green and red indicate decreased and increased expression, respectively. [file Image3.TIF]

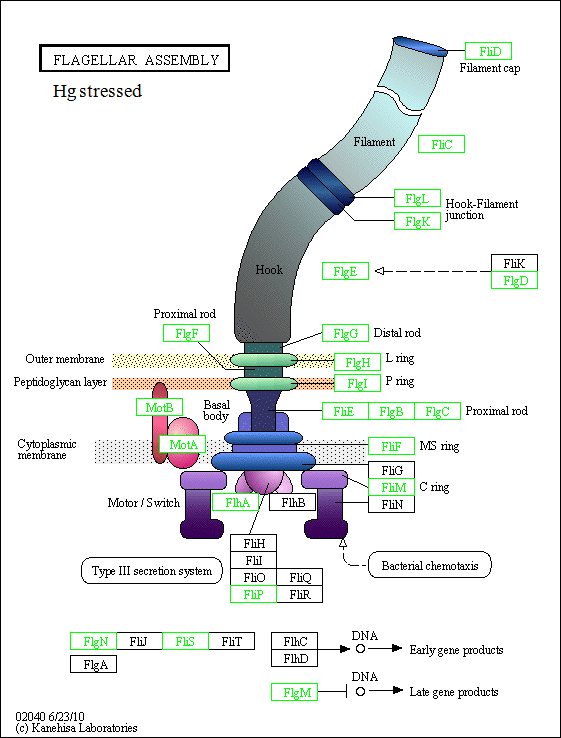

Supplement: Figure S4 — The overall scheme for genes involved in the flagellar assembly pathway that were significantly affected by Hg stress. Green and red indicate decreased and increased expression, respectively. [file Image4.TIF]

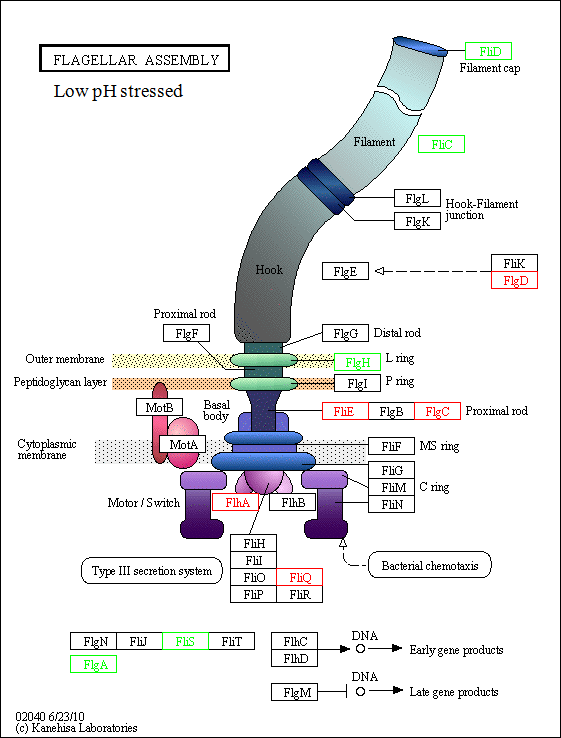

Supplement: Figure S5 — The overall scheme for genes involved in the flagellar assembly pathway that were significantly affected by low pH stress. Green and red indicate decreased and increased expression, respectively. [file Image5.TIF]
