# Supplementary material for: Involvement of the flagellar assembly pathway in Vibrio alginolyticus adhesion under environmental stresses
Source: Front Cell Infect Microbiol. 2015 Aug 12;5:59. doi: 10.3389/fcimb.2015.00059 (PMC4533019; doi:10.3389/fcimb.2015.00059)
Supplement: Supplementary file 6 [file DataSheet1.DOCX]

**Table S1. siRNA Sequence**

| **Target gene** | **siRNA for transient gene silence** |
| --- | --- |
| FliD | F: 5' GGAGAAAGCAUUUGCCGUUTT 3'  R: 5' AACGGCAAAUGCUUUCUCCTT 3' |
| FliC | F: 5' GGUGACCAAUAUCAUGCAATT 3'  R: 5' UUGCAUGAUAUUGGUCACCTT 3' |
| FlgH | F: 5' GCGGUAGAAGGCGAUAAAUTT 3'  R: 5' AUUUAUCGCCUUCUACCGCTT 3' |
| FliS | F: 5' GCGCCUUAUUCAAGGUAAATT 3'  R: 5' UUUACCUUGAAUAAGGCGCTT 3' |
| Negative control | F: 5'-UUCUCCGAACGUGUCACGUTT-3'  R: 5'-ACGUGACACGUUCGGAGAATT-3' |
