# Supplementary material for: Involvement of the flagellar assembly pathway in Vibrio alginolyticus adhesion under environmental stresses
Source: Front Cell Infect Microbiol. 2015 Aug 12;5:59. doi: 10.3389/fcimb.2015.00059 (PMC4533019; doi:10.3389/fcimb.2015.00059)
Supplement: Supplementary file 7 [file DataSheet2.DOCX]

**Table S2. Oligonucelotides used in producing shRNA for stable gene scilencing**

| **Target gene** | **shRNA sequence for** **stable gene silence** |
| --- | --- |
| FliD | F: CTAGGGGAGAAAGCATTTGCCGTTTTTTCAAGAGAAAAACGGCAAATGCTTTCTCCTTTTTTC  R: CCCTCTTTCGTAAAGCCGAAAAAAGTTCTCTTTTTGCCGTTTACGAAAGAGGAAAAAAGCATG |
| FliC | F: CTAGGGGTGACCAATATCATGCAATTTTCAAGAGAAATTGCATGATATTGGTCACCTTTTTTC  R: CCCACTGGTTATAGTACGTTAAAAGTTCTCTTTAACGTACTATAACCAGTGGAAAAAAGCATG |
| FlgH | F: CTAGGGCGGTAGAAGGCGATAAATTTTTCAAGAGAAAATTTATCGCCTTCTACCGCTTTTTTC  R: CCGCCATCTTCCGCTATTTAAAAAGTTCTCTTTTAAATAGCGGAAGATGGCGAAAAAAGCATG |
| FliS | F: CTAGGGCGCCTTATTCAAGGTAAATTTTCAAGAGAAATTTACCTTGAATAAGGCGCTTTTTTC  R: CCGCGGAATAAGTTCCATTTAAAAGTTCTCTTTAAATGGAACTTATTCCGCGAAAAAAGCATG |
