# Supplementary material for: Involvement of the flagellar assembly pathway in Vibrio alginolyticus adhesion under environmental stresses
Source: Front Cell Infect Microbiol. 2015 Aug 12;5:59. doi: 10.3389/fcimb.2015.00059 (PMC4533019; doi:10.3389/fcimb.2015.00059)
Supplement: Supplementary file 8 [file DataSheet3.DOCX]

**Table S3. Primers for QPCR**

| **Gene** | **Primers for QPCR** |
| --- | --- |
| FliD | F: 5' TATCACAGGTATTACAGGCAGTATTCG 3'  R: 5' CCCAACGCACTCATCAAAGC 3' |
| FliC | F: 5' TCCCACAAGGACGCCATAAA 3'  R: 5' CTCCGCACGACTGCCATCTA 3' |
| FlgH | F: 5' GTATTGGCGACATTATTACCGTGA 3'  R: 5' GAGGTTGCCGTTTGCCAGTA 3' |
| FliS | F: 5' CTGTCTATCTATGGATGACGGTGGT 3'  R: 5' TATGAAACTCTGTTGGGATTTGGTC 3' |
| 16S RNA | F: 5'-GGGGAGTACGGTCGCAAGAT-3'  R: 5'-CGCTGGCAAACAAGGATAAGG-3' |
| Gapdh | F: 5'-GATGAGCCAGTTCTTGGATTGC-3'  R: 5'-CCAATGTTGTCGGTTCTTTCGA-3' |
| GyrA | F: 5'-GATTACATTCCTGGTCCTGATTTCC-3'  R: 5'-AGTGCACTGATGCCTTCAACTTTCT-3' |
| LdhA | F: 5'-ATTGATCCGCACGCACTTCT-3'  R: 5'-TCCAATAACCGTAGGTAGGCTGA-3' |
| RecA | F: 5'-GCCAATGGGTCGTATCGT-3'  R: 5'-GCCGCAACTGAGTCAACA-3' |
| RpoD | F: 5'-AATGAGCAGGACTATTACAACCG-3'  R: 5'-ATGTTGATAGAACAGAGTAAGCAGC-3' |
