# Supplementary material for: Involvement of the flagellar assembly pathway in Vibrio alginolyticus adhesion under environmental stresses
Source: Front Cell Infect Microbiol. 2015 Aug 12;5:59. doi: 10.3389/fcimb.2015.00059 (PMC4533019; doi:10.3389/fcimb.2015.00059)
Supplement: Supplementary file 9 [file DataSheet4.DOCX]

**Table S4. Culture conditions of *V. alginolyticus***

| **Cu^2+^ (mg/l)** | 50.0 | 80.0 | - | - | - | - | - | - | - | - |
| --- | --- | --- | --- | --- | --- | --- | --- | --- | --- | --- |
| **Pb^2+^ (mg/l)** | - | - | 100.0 | - | - | - | - | - | - | -- |
| **Hg^+^ (mg/l)** | - | - | - | 50.0 | - | - | - | - | - | -- |
| **pH** | 7.0 | 7.0 | 7.0 | 7.0 | 9.0 | 5.0 | 7.0 | 7.0 | 7.0 | 7.0 |
| **NaCl(g/l)** | 20.0 | 20.0 | 20.0 | 20.0 | 20.0 | 20.0 | 60.0 | 5.0 | 20.0 | 20.0 |
| **Temperature (℃)** | 28 | 28 | 28 | 28 | 28 | 28 | 28 | 28 | 40 | 28 |
